# Assessing the Barriers to Postpartum Tubal Ligation Among Multiparous Women

**DOI:** 10.7759/cureus.27602

**Published:** 2022-08-02

**Authors:** Avir Sarkar, Maninder K Ghotra, Isha Wadhawan, Sonam Jindal, Rinchen Zangmo, Abhik Sarkar

**Affiliations:** 1 Obstetrics and Gynaecology, Employees' State Insurance Corporation (ESIC) Medical College and Hospital, Faridabad, IND; 2 Obstetrics and Gynaecology, All India Institute of Medical Sciences, Delhi, IND; 3 Obstetrics and Gynaecology, Fortis Escorts Hospital, Faridabad, IND; 4 Department of Community Medicine, Rajiv Gandhi University, Bengaluru, IND

**Keywords:** low resource setting, contraception, unmet needs, developing world, postpartum tubal ligation

## Abstract

Background: Unmet demands for postpartum tubal ligation are estimated to be greater than the actual number of tubal ligations done, especially in low-resource settings. Through this study, we therefore assessed the barriers to postpartum tubal ligation in the developing world.

Materials and Methods: It is a prospective cohort study including 3671 multiparous women from northern India. Recruited patients were given survey questionnaires during their antenatal and postpartum period which evaluated the patient-related, healthcare facility-related and social factors which were the determining factors for the unfulfillment of their desire for permanent sterilization.

Results: Out of the recruited population, 1576 women wished to undergo tubal ligation. Following attrition, a total of 1024 were followed up prospectively. Of them, sterilization was successfully done only in 309 (30.18%) participants. A large proportion of mothers had their demand unmet (715 mothers; 69.82%). Out of them, 505 (70.63%) women stated that tubal ligation was not done because they did not deliver by Caesarean section. Insufficient counselling regarding tubal ligation was quoted by 325 (45.45%) mothers. Majority of the participants denied tubal ligation as they were not given enough information about the procedure (589 participants; 82.38%). While 568 (79.44%) mothers had changed their mind after delivery, 257 (35.94%) thought that their health was not fit to undergo the procedure and 213 (29.79%) mothers wanted more children in the future. We found that there was strong opposition from their male partners (56.78%).

Conclusion: Postpartum tubal ligation is of paramount importance in women with completed families, especially in developing countries. This prospective study addressed the barriers to tubal ligation, which would help combat future unintended pregnancies.

## Introduction

Tubal ligation is done after 10% of all deliveries, and the frequency is much higher following caesarean sections [1,2]. Despite higher rates of tubal sterilization, many women who desire the procedure are not able to get it done after delivery prior to hospital discharge [3]. Studies conducted in various hospital settings in the western world have shown that 31-48% of requests for postpartum tubal ligation are not met [4,5]. The common barriers to postpartum tubal ligation in our low-resource settings are lack of knowledge and reluctance by the male partner and family members. Most public sector hospitals are overburdened with patients and so there is hardly any time for antenatal counselling regarding postpartum contraception. So, women are unaware of the options of family planning. In the traditional age-old thinking, women do not generally comply with artificial methods of contraception. So, it is a real challenge to counsel the family members to accept permanent methods of contraception.

Unmet demands for postpartum tubal ligation are typically assessed in retrospective studies by comparing the tubal ligation request forms with the records of procedures performed. The present study focused on identifying this disparity between willingness to have tubal ligation and actual procedure performed through a prospective analysis. This study identified the demands for postpartum tubal ligation among the multiparous population to ascertain the barriers encountered after delivery. In this way, an estimate of the correct proportion of unmet demands for postpartum contraception was assessed.

## Materials and methods

This was a prospective observational study of pregnant women conducted at the antenatal clinic of ESIC Medical College and Hospital for a duration of six months. Informed consent was obtained from all participants. All procedures in the study involving human participants were performed in accordance with the ethical standards of the Institutional Ethics Committee of ESIC Medical College and Hospital (approval IEC-203/21) and with the 1964 Helsinki Declaration and its later amendments or comparable ethical standards. Consecutive consenting women were recruited into the study and a questionnaire was used to obtain information about the sociodemographic and reproductive characteristics of the participants. The willingness to accept postpartum tubal ligation was then asked from the respondents. Desire for postpartum contraception was asked of all multiparous women between 22 to 49 years of age at the time of routine antenatal visit during the third trimester. Due to large patient load, antenatal women were counselled only in the third trimester. Eligible women who desired to opt for postpartum tubal ligation as the contraceptive of choice were recruited into the study. Criteria for exclusion consisted of women with no living child more than one year old, women who were not in a sound state of mind so as to understand the full implications of tubal sterilization, women whose partner had already undergone vasectomy as a method of permanent contraception and those who were not willing to give valid written consent. A survey questionnaire in the vernacular language was provided to all the participants fulfilling the inclusion criteria. This set of questions assessed their knowledge of the various methods of contraception available free of cost by the Government of India.

During the time of delivery, tubal sterilization was avoided in women giving birth to babies with gross congenital malformations or babies who did not cry immediately, thereby needing resuscitation. These women were later excluded from the study. At six weeks to three months postpartum, when they came for postpartum visit with their babies, they were asked to answer a second set of questions that assessed whether their demand for tubal ligation was fulfilled or not. If the desire for tubal sterilization was fulfilled, they were asked about the method by which the procedure was done. If not, this questionnaire evaluated a detailed assessment of the cause for unfulfillment of this desire. This questionnaire was obtained from the pretested set of questions used in the study conducted earlier by Potter et al. in Texas, USA [3]. All data were tabulated in a Microsoft Excel worksheet (Microsoft, Redmond, WA, USA) and statistical analysis was performed by SPSS version 22.0 (IBM Corp., Armonk, NY, USA). Rates and proportions were calculated for categorical variables. Bivariate analysis using independent T-test and Fischers exact was used to compare the demographic features between women undergoing sterilisation and those who did not. Additional multivariate logistic regression was used to identify factors that were associated with failure of undergoing tubal sterilisation. 

## Results

**Figure 1:**
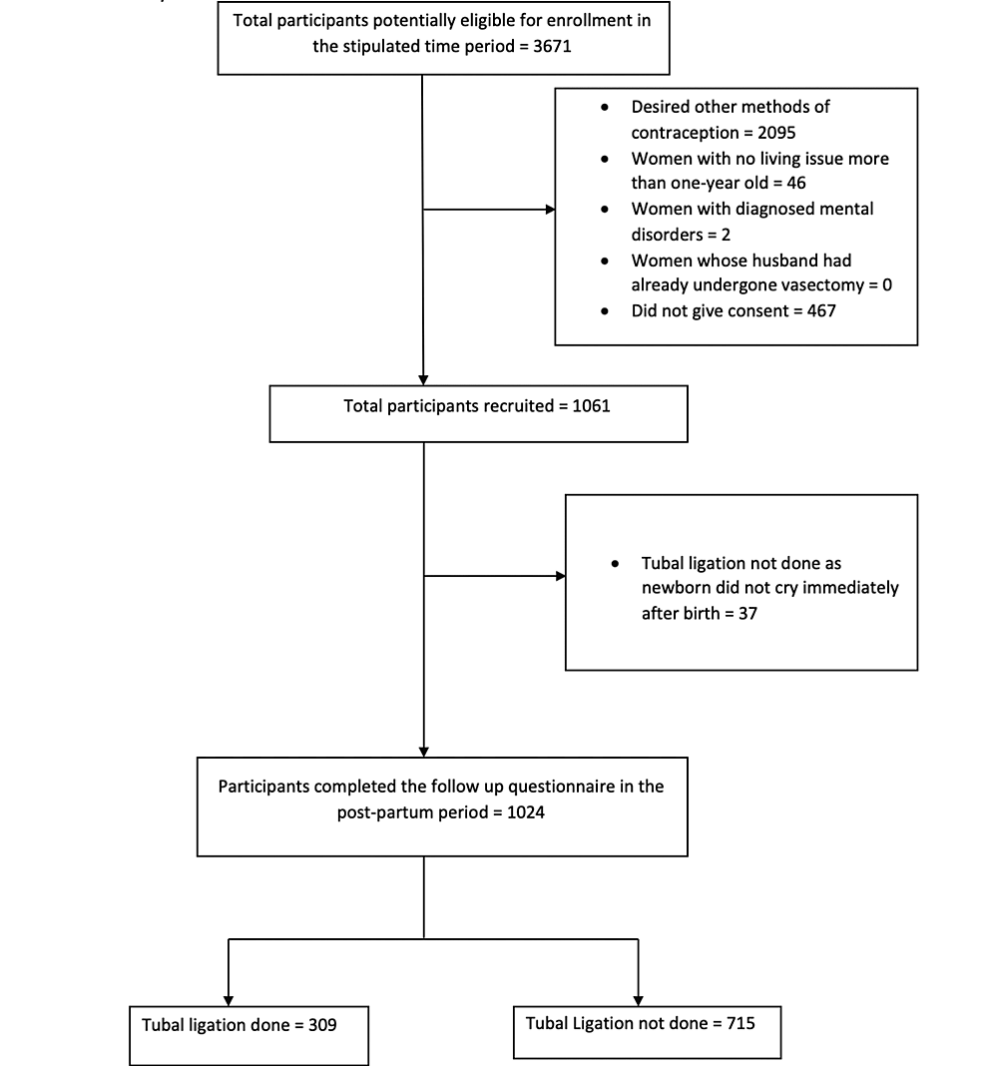
Flowchart showing the number of participants recruited and followed prospectively during the study time frame

A total of 3671 multigravida were potentially eligible for enrolment during the study time frame. Of them, 2095 women desired other methods of contraception when given various options of choice. Fifteen hundred seventy-six women wished to undergo tubal ligation as a permanent method of contraception. But 515 women had to be excluded from the study (46 women had no living child more than one year of age, two women were diagnosed with schizophrenia and could not give valid consent, and 467 women refused to participate in the study and most of them were reluctant to follow up for postpartum visits at the same institute). Thus, a total of 1061 mothers were recruited into the study (Figure 1). They were asked to fill out the antenatal questionnaire. Prospective follow-up was done and they delivered at the same hospital. Seven hundred forty-eight (748; 73.05%) women delivered vaginally while 313 (26.95%) delivered through a lower segment Caesarean section (LSCS). The obstetrician decided not to perform tubal ligation in 37 mothers as the newborn did not cry immediately after birth. Thus, only 276 (26.95%) women in the LSCS group were included in the analysis. At six weeks to three months follow-up visits, 1024 mothers completed the follow-up questionnaire. Of them, tubal sterilization was successfully done in 309 (30.18%) participants. A large proportion of mothers had not undergone tubal ligation (715 mothers; 69.82%) and their desire for contraception remained unmet (Figure 1).

Most of the participants were young multiparous (25.2 ± 3.4 years) with a median parity of two. None of the participants were insured for tubal ligation under any government scheme. Most of the women followed Hinduism (967 women; 94.43%). The majority of them were educated from 6th to 12th standard (798 women; 77.92%) (Table 1). According to Modified Kuppuswamy Scale, the majority of the women belonged to the lower-middle socio-economic class (429 women; 41.89%). A bivariate analysis compared the baseline parameters between the women undergoing tubal ligation with those who could not (Table 2). It was found that there were significant differences in the mean age, parity, education, socioeconomic status and religion between the two groups (p value of 0.05, 0.025, 0.016, 0.012 and <0.001 respectively). Insurance for tubal ligation was not significantly different between the groups (p value of 0.9).

**Table 1:** Demographic features of the population under study

Assessments	Parameters	Numbers	Percentage
Parity	Two	814	79.49%
	Three	173	16.89%
	Four	35	3.42%
	Five	2	0.20%
Whether insured for TL under any government scheme	Insured for Tubal Ligation	0	0
	Not insured	1024	100%
Religion	Hinduism	967	94.43%
	Islam	51	4.98%
	Sikhism	6	0.58%
Education Status	Uneducated	36	3.51%
	Up to 5th standard	78	7.61%
	6th to 12th standard	798	77.92%
	Graduate	112	10.93%
Socio-Economic Status	Lower	90	8.78%
	Upper Lower	307	29.98%
	Lower Middle	429	41.89%
	Upper Middle	126	12.31%
	Upper	72	7.03%

**Table 2:** Comparison of demographic features of the population under study

Assessments	Parameters	Women not undergoing Tubal Ligation (n=715)	Women undergoing Tubal Ligation (n=309)	P value
1. Mean age (in years)		25.01	25.72	0.05
2. Parity	Two	620(86.71%)	194(62.78%)	0.025
	Three	90(12.59%)	83(26.86%)	
	Four	5(0.70%)	30(9.71%)	
	Five	0(0.00%)	2(0.65%)	
3. Education status	Uneducated	29(4.06%)	7(2.27%)	0.016
	Up to 5th standard	63(8.81%)	15(4.85%)	
	6th to 12th standard	570(79.72%)	228(73.79%)	
	Graduate	53(7.41%)	59(19.09%)	
4. Socio-economic status	Lower	397(55.52%)	0(0.00%)	0.012
	Middle	257(35.94%)	298(96.44%)	
	Upper	61(8.53%)	11(3.56%)	
5. Religion	Hinduism	658(92.02%)	309(100%)	<0.001
	Islam	51(7.13%)	0(0.00%)	
	Sikhism	6(0.84%)	0(0.00%)	
6. Whether insured for tubal ligation under any government scheme	Yes	0(0.00%)	0(0.00%)	0.9
	No	715(100%)	309(100%)	

On applying multivariate analysis, maternal age, parity, education and socioeconomic status were found to be associated with failure of undergoing tubal ligation in the study population (adjusted odds ratio of 1.070, 3.747, 1.576, 4.834 and p values of 0.007, <0.001, 0.002, <0.001 respectively) (Table 3). Odds ratio could not be calculated for religion as there were no participants (zero values) in Islam and Sikhism among women who underwent tubal sterilisation. 

**Table 3:** Multivariate analysis showing the factors associated with failure of undergoing tubal ligation in the study population and their respective odds ratios

Parameters	Unadjusted Odds Ratio	Adjusted Odds Ratio (95% CI)	P value
Age	1.097	1.070 (1.02, 1.12)	0.007
Parity	4.229	3.747 (2.75, 5.10)	<0.001
Education status	0.384	1.576 (1.18, 2.10)	0.002
Socio-economic status	54.076	4.834 (3.61, 6.47)	<0.001

Out of 1024 mothers who completed the postpartum follow-up, 309 (30.18%) had undergone tubal ligation. Of them, 244 (78.96%) had tubal ligation done concurrent with LSCS, while 53 (17.16%) and 12 (3.88%) women underwent tubal ligation through mini-laparotomy and laparoscopic routes respectively. On the other hand, out of the 715 (69.82%) women in whom tubal ligation was not done, participants reported to have several reasons for unfulfillment of their desire (Table 4). Hospital system barrier was present in 530 (74.12%) participants. There was a problem with consent forms in 25 (3.49%) cases. Tubal ligation was not done in 505 (70.63%) mothers because they were not delivered through the Caesarean route. Provider barrier was present in 325 (45.45%) mothers where the doctor or staff could not discuss with them in detail regarding tubal ligation after delivery. Patient barrier in the form of denial because their child was too young for them to undergo sterilization was seen in 24 (3.36%) mothers. Maximum participants denied ligation as they were not given enough information about the procedure (589 participants; 82.38%). Some mothers (568, 79.44%) had changed their mind after delivery, 257 (35.94%) mothers thought that their health was not fit to undergo the procedure and 213 (29.79%) mothers wanted more children in the future. Society was also a hurdle to a permanent method of contraception. The majority of the women could not fulfill their demand for ligation as they reported that there was strong opposition from their husbands (406, 56.78%). In 148 (20.69%) women, other family members also opposed tubal ligation. Among 45 (6.29%) women, tubal ligation was not an acceptable method of contraception in their society (Table 4). 

**Table 4:** Causes for unfulfillment of the desire for postpartum tubal ligation

Barriers to postpartum TL	Parameters assessed	Numbers	Percentage
Hospital System Barrier	Problem with Consent forms	25	3.49%
	Because Caesarean section was not done	505	70.63%
	OT was not available	0	0
Provider Barrier	Doctor didn't discuss with me	325	45.45%
	Doctor was not willing to do tubal ligation	0	0
Patient Barrier	I have changed my mind	568	79.44%
	Health is not fit for tubal ligation	257	35.94%
	Not given enough information	589	82.38%
	I want more children	213	29.79%
	I feel my child is too young	24	3.36%
Family and peer pressure	Partner opposed	406	56.78%
	Other family member opposed	148	20.69%
	Lack of social acceptance	45	6.29%

## Discussion

India is the second most densely populated country in the world with a population of 1.39 billion. Permanent contraception is a common method of fertility control worldwide [6,7]. Even though permanent contraception can be offered anytime, we believe the postpartum period serves as a golden opportunity as the woman is in the hospital setting and comes for revisits also. Women have the solace of a complete family after delivery, lower failure rates and most importantly prevention of unintended pregnancy during the postpartum period. However, permanent contraception decision-making can be complex, in the sense that the decision-making requires a comprehensive review of available options and relevant outcomes, benefits as well as potential risks of the procedure. Thus an informed choice that is aligned with each patient’s reproductive goals should be met. In our study, we have evaluated those critical factors involved in postpartum tubal ligation related to the patient, their family, health care provider and hospital infrastructure. The majority population in our study belonged to the upper-lower and lower-middle class of socioeconomic status. Speaking of the education qualification, which is an important element for the knowledge and acceptance of contraception, the majority of our population have been educated up to 12th grade. A systematic review by Pazol et al. provides clear evidence that a wide range of educational tools can effectively increase client knowledge and acceptance [8].

In the index study, the lion’s share of the failure of provision of tubal ligation was patient-related factors. Many women who were motivated for permanent sterilisation during their antenatal period rejected tubal ligation because they changed their minds. Many women changed their decision about the permanent nature of sterilization following delivery. They had variable explanations regarding their personal decision to cancel or postpone the procedure. In another subset of women, last-minute misgivings were related to ambivalence regarding desired family size, concerns about poor neonatal outcomes in case of prematurity, and maternal comorbidities. Similarly, Gilliam et al. evaluated these barriers of women’s perspective to postpartum sterilization and stressed the importance of counselling and decision-making [9]. In the study by Seibel-Seimen et al., a retrospective study on evaluation of factors predicting failure of postpartum tubal ligation following vaginal delivery, out of the 135 women who requested sterilization only 56% received the desired procedure [10]. In our study 715 mothers (69.82%) did not undergo tubal ligation. The common reason for the above was that the women were unwilling to undergo an additional surgical procedure for contraception after vaginal birth. In a retrospective study by Kouman et al. the authors stressed in the context of developing countries tubal ligation through mini-laparotomy should be promoted in family planning programs [11]. Sterilisation through mini-laparotomy should be promoted in national family planning programs. We strongly believe that this facet when addressed has the potential of a game changer in the play of postpartum sterilization.

A woman's reason for unfulfilled postpartum tubal ligation request can be categorized as “provider influence” when she changed her mind about undergoing the procedure after being admitted for delivery. Our in-depth interviews revealed that some women elected to cancel the procedure when providers shared presumably new and/or inconsistent information about the surgical procedure and its associated risks or when they felt enough information was not given by the providers and hospital staff. These reasons for denial suggest an integral role of provider influence in postpartum sterilization decision-making and outcomes among the patient population. Especially in a developing country like India where the labor ward is buzzing with umpteen patients but limited healthcare staff, it is practically arduous for the doctor to give quality time to counselling the couple. Appointing an exclusive counsellor for this purpose, in our opinion, could overcome this issue and aid the couple in assisting their decision on postpartum contraception.

Traditional practices, cultural expectations, family and peer pressure are strongly anchored in an individual regarding fertility as well as contraception. In our study 45 patients ascertained that sterilization practice was not socially acceptable. Srikanthan et al. conducted a study on the stereotypical religious, social, and cultural characteristics of women seeking contraception and stressed the need for healthcare providers to recognize different value systems that may influence contraception in couples of different faiths [12]. Opposition from the partner and the family members has been majorly influencing a woman's decision for contraception especially if it is permanent contraception. The lack of sense of autonomy in women for decision-making has since time immemorial strongly affected the acceptance of postpartum sterilization. Prompt counselling of the women and the partner and also an integrated approach involving the family sentiments can provide a holistic solution to this compound hitch. Additional measures should be undertaken to ensure that patient requests for postpartum tubal ligation are fulfilled. The strength of our study lies in the very fact that it is the first prospective analysis to assess the unmet demand for tubal ligation in the postpartum period in a huge cohort of the population hailing from a low resource setting in India. No prospective data with such a large number of participants has been published from low-income countries till now.

Our study had a few limitations. It was a single center study. Considering the huge patient load, no separate family planning counselor could be appointed for effective contraceptive counseling in the antenatal period. It was also not possible to devise strategies to improve the low prevalence of postpartum tubal ligation in our low resource set-up. 

## Conclusions

Most women desire to limit future pregnancies during the antenatal period. However, lack of knowledge and difficulty in accessing the family planning services in our centre are the major limitations in this field. It is the time when government policies need to be strengthened through public propaganda and awareness schemes reaching to the farthest of the rural areas. Addressing the barriers to this issue would help combat future unintended pregnancies and hence the population crisis.
